# A synthetic biology toolkit for rationally designing genetic circuits in *Acinetobacter baumannii*

**DOI:** 10.3389/fsysb.2025.1668595

**Published:** 2026-01-15

**Authors:** Sara Letrari, Lisa Faccincani, Stefano Intini, Ilgin Ertan, Tommaso Varaschin, Francesca Galiazzo, Marco Costanzo, Giorgia D’angelo, Valentina Del Giudice, Luca Guarnieri, Alex Martini, Asia Picchi, Chiara Ravazzolo, Niccolò Venturini Degli Esposti, Chiara Zanin, Livio Trainotti, Cristiano De Pittà, Claudia Del Vecchio, Ignazio Castagliuolo, Massimo Bellato

**Affiliations:** 1 UniPadua-IT 2023 iGEM Team, University of Padova, Padova, Italy; 2 Department Molecular Medicine, University of Padova, Padova, Italy; 3 Department Information Engineering, University of Padova, Padova, Italy; 4 Department Medicine, University of Padova, Padova, Italy; 5 Department Biology, University of Padova, Padova, Italy

**Keywords:** *Acinetobacter baumannii*, genetic circuit, BioBrick, CRISPRi, gene expression, synthetic and systems biology

## Abstract

**Introduction:**

Antimicrobial resistance (AMR) poses a severe global health threat, with *Acinetobacter baumannii* among the critical AMR priorities highlighted by World Health Organization (WHO). This Gram-negative pathogen exhibits intrinsic resistance traits, exceptional environmental persistence, and high genomic plasticity, harboring resistance islands.

**Methods:**

To combat AMR through synthetic biology, this study characterizes a library of BioBrick parts to be adopted in *A. baumannii* engineering and develops a modular CRISPR interference (CRISPRi) platform.

**Results:**

Key components were characterized, including two plasmid vectors, a library of inducible and constitutive promoters, and a CRISPRi-mediated repression system; for the latter, a testbed for biofilm-related genes implicated in the downregulation of antibiotic resistance is also provided.

**Discussion:**

By enabling tunable transcriptional control through the characterized promoters and ensuring the ability to downregulate gene expression via CRISPRi, this synthetic biology toolkit lays the foundation for the rational design of genetic circuits to study and counteract AMR in *A. baumannii*. The modular platform here characterized provides a valuable resource for the iGEM community to advance functional genomic approaches against this alarming global health challenge.

## 1 Introduction

Antimicrobial resistance (AMR) is recognized as a major global health threat. It occurs when microorganisms acquire mechanisms—either intrinsic or acquired—that render them non-susceptible to antimicrobial agents, leading to infections with elevated morbidity and mortality (Mancuso et al., 2021; Christaki et al., 2020). In 2019, the World Health Organization (WHO) declared AMR among the top ten global public health threats (World Health Organization, 2021), with a substantial economic burden due to prolonged hospitalizations and increased demand for treatment.

Currently, AMR is responsible for approximately 700,000 deaths annually worldwide. According to the Review on Antimicrobial Resistance, this figure could rise to 10 million by 2050 without effective intervention (O’Neill, 2016). In the EU/EEA, the European Antimicrobial Resistance Surveillance Network (EARS-Net) reported over 670,000 AMR infections and 33,000 AMR-related deaths annually, with associated healthcare costs estimated at €1.1 billion (Montalti et al., 2022). The overuse and misuse of antibiotics in human medicine and agriculture significantly contribute to the spread of AMR. In livestock farming, antibiotics are widely used to promote growth and improve feed efficiency, thus facilitating the selection and spread of resistant bacterial strains that can enter the food chain and reach humans (Pendleton and Davidson, 2017; Cheng et al., 2019). Resistance mechanisms can be intrinsic—driven by structural or physiological traits like outer membrane impermeability or biofilm production—or acquired—through horizontal gene transfer or spontaneous mutations (Pendleton and Davidson, 2017; Prestinaci et al., 2015; Holmes et al., 2016).

*Acinetobacter baumannii*, a Gram-negative, nonmotile, aerobic coccobacillus, is among the ESKAPE pathogens—*Enterococcus faecium*, *Staphylococcus aureus*, *Klebsiella pneumoniae*, *A. baumannii*, *Pseudomonas aeruginosa*, and *Enterobacter* spp.—highlighted by the WHO as critical AMR priorities (Mancuso et al., 2021). *A. baumannii* is specifically implicated in severe nosocomial infections such as pneumonia, bacteremia, meningitis, and urinary tract infections, especially in immunocompromised individuals. Its clinical relevance is attributed to intrinsic resistance traits, exceptional environmental persistence (e.g., tolerance to desiccation, UV, detergents, and disinfectants), and high genomic plasticity, with strains harboring up to 20 resistance- and virulence-associated genomic islands (Christaki et al., 2020; Pendleton and Davidson, 2017; Barbe et al., 2004; Antonelli and Rossolini, 2023).

To address gene regulation in *A. baumannii* and explore therapeutic strategies, CRISPR interference (CRISPRi) offers a programmable system for transcriptional repression. CRISPRi uses a catalytically inactive Cas9 (dCas9) guided by a synthetic single-guide RNA (sgRNA) to bind target DNA without cleavage, thereby blocking transcription (Qi et al., 2013; Larson et al., 2013; Ghavami and Pandi, 2021). sgRNA mimics the crRNA–tracrRNA complex; it consists of a 20-nt target-specific spacer, a 42-nt scaffold for dCas9 binding, and a 40-nt terminator. This simplified design bypasses native CRISPR RNA maturation pathways. Transcriptional repression efficiency is higher when targeting promoter regions as opposed to coding sequences due to more effective obstruction of transcription initiation versus elongation (Ghavami and Pandi, 2021). Although CRISPRi has been extensively validated in *Escherichia coli*—also in our laboratory (Huang et al., 2021; Bellato et al., 2022; Frusteri Chiacchiera et al., 2025)—its application in *A. baumannii* requires system-specific optimization due to the lack of modular, standardized, and pre-characterized genetic parts for the synthetic biology community planning to work on this specific species.

For the delivery of CRISPRi components into *A. baumannii*, the pWH1266 shuttle vector, a hybrid plasmid derived from *Acinetobacter calcoaceticus* (pWH1277) and *E. coli* (pBR322), is the most common, despite not being compliant with standard assembly diffused in the iGEM community (e.g., RFC [10] (Knight and Thomas, 2007). It contains replication origins for both hosts and shows copy numbers of ∼ 56–70 per cell in *A. baumannii* and *E. coli*, respectively (Lucidi et al., 2018; Wang et al., 2019). The pSGAb-km variant, in which the β -lactam resistance gene (*bla*) was replaced by a kanamycin cassette to ensure compatibility with resistance markers in experimental setups, is used in this study. This vector has been successfully used in previous CRISPR-based genetic engineering applications in *A. baumannii*. For example, Wang et al. (2019) combined pSGAb with pCasAb to construct a dual-plasmid genome-editing system employing CRISPR-Cas9 cleavage and native *A. baumannii* RecAb recombination machinery for precise gene deletions, insertions, and point mutations. Brychcy et al. (2023) developed a functional CRISPRi system in *A. baumannii* using plasmids derived from pWH1266. The system included a plasmid carrying an anhydrotetracycline (aTc)-inducible dCas9, constitutively expressed sgRNAs targeting fluorescence reporters (eGFP and mCherry), and fluorescence reporter genes; a second control plasmid with the same reporters but non-targeting sgRNAs was also included in the study. CRISPRi-mediated repression was observed only in cells harboring the former circuit upon ATc induction, with a dose-dependent decrease in fluorescence, validating the possibility of a CRISPRi approach in *A. baumannii*.

Building upon such studies and an exploratory work from the Uni-Padua-IT iGEM Team (2023), this study aims to combat AMR through synthetic biology by developing a modular CRISPRi platform that is simple to use for the iGEM community for engineering and studying *A. baumannii*. In this study, the characterization of a (i) dual-plasmid system, (ii) library of inducible and constitutive promoters, and (iii) CRISPRi repression system of biofilm-related genes–key mediators of antibiotic resistance is provided.

It is worth noting that while Wang et al. (2019) have already developed powerful CRISPR-based genome-editing and base-editing platforms in *A. baumannii*, these approaches are primarily suited for creating stable knockouts or precise sequence modifications. Furthermore, although such strategies may be sufficient for antimicrobial resistance research in many cases, they can also facilitate the emergence of new mutations and consequent resistance. On the other hand, in addition to avoiding DNA damage and mutation onset, CRISPRi offers complementary and unique advantages that cannot be achieved through permanent editing. Specifically, CRISPRi allows for the conditional knockdown of essential genes that cannot be deleted without affecting cell viability, enabling the study of essential pathways in *A. baumannii*. Additionally, the ability to multiplex multiple sgRNAs in a single system permits the coordinated regulation of several genes simultaneously, which is critical for studying combination resistance mechanisms or complex regulatory networks. Finally, CRISPRi is inherently suitable for scalable, high-throughput functional screening of gene libraries, as repression is reversible, tunable, and does not produce permanent genomic scars. These features make CRISPRi an essential tool for functional genomics in *A. baumannii*, complementing existing genome- and base-editing methods and expanding the toolkit available for AMR research.

By introducing the characterization of novel parts described here, our system lays the foundation for rational genetic circuit design and functional genomics in AMR pathogens.

## 2 Materials and methods

### 2.1 Strains, media, and cultures

The *E. coli* DH5 α (Thermo Fisher Scientific) strain was used as a host for cloning, while *A. baumannii* 2208 and 5377 (ATCC # 19,606 and # 17,978 strains, respectively) were used to characterize parts and circuits. *E. coli* strains were transformed by heat shock according to the manufacturer’s instructions, while *A. baumannii* was electroporated optimizing the protocol reported in Biswas and Rather (2019). In brief, 1 μ L (∼20n5) DNA was added into 50 μ L of freshly prepared electro-competent cells; an electric pulse (1800 V, 100 Ω , 25 μ F, 6 m) was applied into a 1-mm cuvette, followed by cell recovery grown for 1 h at 37 °C in 900 μ L of SOC medium (New England Biolabs - B9020S). For general bacterial and plasmid propagation, LB broth (NaCl, 10 g/L; tryptone, 10 g/L; yeast extract, 5 g/L) was used. Antibiotics were added to maintain plasmids in recombinant strains: ampicillin (100 mg/mL), carbenicillin (100 mg/mL), kanamycin (50 mg/mL), or chloramphenicol (12.5 mg/mL), as required by the vector backbones pSB1A2, pSB3K3, pSB4C5 (Shetty et al., 2008), pSGAb-km, and pME6032. *E. coli* was grown overnight at 37 °C, 150 rpm, while *A. baumannii* was grown at 37 °C, 200 rpm. Growth on plates was performed on selective LB Agar (15%) plates. Long-term stocks were prepared for all strains by mixing 750 μ L of a saturated culture with 250 μ L of 80% glycerol and storing them at −80 °C.

### 2.2 Cloning procedure

A list of the plasmids used in this study is reported in Supplementary Table S1, and the maps and details are available as entries in the Registry of Standard Biological Parts (2025). All plasmids used in this study were constructed using the BioBrick Standard Assembly (Knight, 2003), Gibson assembly, and conventional molecular biology techniques, ensuring compatibility with RCF [10]. In the former case, as a result, standard DNA junctions (TACTAG upstream of coding sequences, TACTAGAG otherwise) were present between assembled parts. The basic or composite parts used for DNA assembly were retrieved from the MIT Registry 2008–2011–2023 DNA Distribution or gifted from the BMS Lab, University of Pavia, Italy (Magni, 2025). The desired plasmids were purified with the QIAprep Spin Miniprep purification kits (Qiagen), digested with Standard BioBrick Restriction Enzymes (New England Biolabs), analyzed in a 1% agarose gel, and ligated with T4 Ligase (Thermo Fisher Scientific) overnight at 16 °C. The ligation products were chemically transformed into competent *E. coli* DH5 α .

Colonies carrying the ligated products were screened using Sanger sequencing, which was performed via DNA analysis services from BMR Genomics (Padova, Italy) and Eurofins Genomics Germany GmbH (Ebersberg, Germany). Oligonucleotides for mutagenesis and novel synthetic parts were obtained from Merck KGaA (Darmstadt, Germany) and GenScript Biotech Corporation DNA synthesis service (Nanjing, China).

#### 2.2.1 pSGAb-derived compatible plasmids

The plasmid pSGAb-km was purchased from Addgene (Plasmid #121999). To comply with the BioBrick standard, an intermediate vector was generated by digesting a 118-base-pair synthetic double-stranded DNA fragment—containing a BioBrick-compatible cloning site (Supplementary Material)—and the pSGAb-km plasmid with HindIII and SacI (Thermo Fisher Scientific). To obtain the final pSGAbi construct, the *E. coli* high-copy origin of replication present in the pSGAb-km vector was replaced with the medium-copy p15A origin. This modification was performed using the Gibson Assembly NEBuilder® HiFi DNA Assembly Cloning Kit (New England Biolabs).

#### 2.2.2 Promoter library

A set of inducible and constitutive gene expression systems was cloned, electroporated, and tested in *A. baumannii*, driven by different inducible or constitutive promoters: the HSL-inducible system, the IPTG-inducible system, an aTc-inducible system, and a library of 13 different promoters from the iGEM Anderson library. Constructs were structured through digestion and ligation, ensuring compatibility with the RFC [10] standard assembly. The pSGAbi_J23XX_RFP plasmid set was structured by digesting the BBa_J23XX set of promoters with downstream-encoded red fluorescent protein (RFP) with EcoRI and PstI, to extract the constitutive expression cassettes; these were then inserted into the pSGAbi plasmid. All 13 resulting pSGAbi_J23XX_RFP carried a cassette of RFP expression (BBa_E1010 with ribosome binding site BBa_B0034 and transcriptional terminator BBa_B0015) expressed by the different Anderson promoters.

The pSGAbi-pLuxR plasmid was constructed by digesting both pE47, which contains the HSL-inducible system controlling RFP expression, and pSGAbi with EcoRI and PstI, and then cloning the HSL system into the shuttle plasmid. The aTc-inducible system was obtained as the HSL system by digesting both pSB4C5_aTc and pSGAbi to insert the aTc-inducible system into pSGAbi. Plasmid pSB4C5_aTc is a gift from the BMS Lab (University of Pavia, Italy) and is composed of a pSB4C5 backbone bearing as a BioBrick-compatible insert the BBa_parts: J23118 − B0031 − C0040 − B0015 − R0040 − I13507. The IPTG-inducible system was structured first by digesting one of the pSBJ23XX plasmids with SpeI and PstI to extract the RFP expression cassette without the promoter and then cloning it inside Y32_pLac, digested with SpeI and PstI, to obtain the inducible RFP expression Y32_pLac_RFP driven by pLac. This new plasmid was then digested with EcoRI and PstI to extract the IPTG-inducible system and cloned into pSGAbi as described for the other inducible systems.

#### 2.2.3 CRISPRi system

The dCas9 sequence from the pSB3K3_dCas9 was mutagenized via PCR to eliminate the RCF [10]-forbidden EcoRI restriction. The Q5® Site-Directed Mutagenesis Kit (New England Biolabs) was used according to the manufacturer’s instructions. In brief, mutagenesis was performed by purifying the template plasmid DNA and using it in a PCR reaction with the Q5 polymerase. We ran 5 μ L of the PCR product in a 1% agarose gel, and 1 μ L was treated and enriched with the KLD buffer (New England Biolabs) for 5 min at room temperature. The resulting mixture was directly transformed into competent cells, which were then plated on LB agar plates and incubated overnight at 37 °C.

The pSGAbi_CRISPRi was constructed by assembling the dCas9 gene (isolated from pSB3K3_dCas9), the HSL-inducible sgpTet element (from pAEgpTet), and the I13521 red fluorescent reporter gene (under the pTet promoter, from pSB1A2_I13521). The CRISPRi plasmid harboring the nonspecific guide, *pSGAbi_CRISPRi_asp*, was generated using the Directed Site Mutagenesis Kit (New England Biolabs) by mutagenizing the sgpTet gRNA sequence with the sgpLac, as described below.

The three plasmids carrying the CRISPRi system targeting the *OmpA* gene, along with a nonspecific system used as control, were constructed through a series of cloning steps. First, an intermediate plasmid was generated by digesting pSGAbi and the *dCas9* gene from pSB3K3_dCas9mut, both with EcoRI and PstI, thus allowing insertion of the *dCas9* gene into pSGAbi. To generate the final plasmids targeting *OmpA*, two different single-guide RNAs (sgRNAs) were cloned, either individually or in combination. For pSGAbi_sg157, the intermediate was digested with EcoRI and XbaI, and the sg157 guide was digested with EcoRI and SpeI. For pSGAbi_sg211, the same intermediate was digested with EcoRI and XbaI, and the sg211 guide was digested with EcoRI and SpeI. Finally, to create the dual-guide plasmid pSGAbi_2X, the pSGAbi_sg211 plasmid was digested with EcoRI and XbaI, and the sg157 fragment, obtained from pSGAbi_sg157 digested with EcoRI and SpeI, was ligated into it. Lastly, the aspecific pSGAbi_OmpA_asp control was obtained via PCR mutagenesis, starting from the plasmid carrying sg157.

#### 2.2.4 Two-plasmid system plasmid

In addition to the optimized pSGAbi backbone, pME6032 was chosen as a complementary shuttle vector for *A. baumannii* (Heeb et al., 2002). This plasmid was edited to create a new shuttle vector compatible with RFC [10] standards. Two modifications were performed: first, to change the antibiotic resistance, and then to insert BBa_I13521 into the plasmid, flanked by the sequences of Prefix and Suffix from RFC [10]. Both modifications were performed using the Gibson Assembly method. pME6032 plasmid contains the gene encoding for tetracycline resistance, *TetR*, making it incompatible for use in *A. baumannii*; therefore, it was changed with the beta-lactamase resistance gene *BlaR* via divergent PCR mutagenesis.

In brief, pME6032 was amplified in PCR, excluding the *tetR* gene, while the *BlaR* sequence was amplified in PCR from the pTZ19R (Addgene Plasmid # 4468) plasmid. The two fragments were purified using Promega’s Wizard® SV Gel and PCR Clean-Up System (A9282) and then assembled with GeneArt™ Gibson Assembly HiFi Master Mix (A46627) (Scientifics, 2025), following the manufacturer’s protocol, to create plasmid pME6032_blaR. The latter was, in turn, transformed inside *A. baumannii*. Upon verifying the maintenance of the plasmid in the target host cells on selective medium, pME6032_blaR was subsequently edited to insert the cassette I13521 flanked by the sequences of prefix and suffix for being compliant with RFC [10] standard; PCR amplification was performed to remove a small backbone portion carrying an undesired EcoRI cutting site to avoid any possible interference with further cloning procedures, while BBa_I13521 was amplified from a plasmid with backbone pSB1A2, including the flanking portions of prefix and suffix. The two fragments were purified using Promega’s Wizard® SV Gel and PCR Clean-Up System (A9282) and then assembled using NEBuilder® HiFi DNA Assembly Cloning Kit (E5520S) following the manufacturer’s guidelines and protocols, creating plasmid pME6032_I13521.

Primers used in this work are reported in Supplementary Table S2.

#### 2.2.5 sgRNA design and construction

All sgRNAs used here were designed via the Benchling CRISPR tool Benchling Inc., (2025), setting a guide length of 20 nucleotides, GCA_00005845.2 (*E. coli* K12) or GCA_002136865-ER53 (*A. baumannii*) as reference genomes, and using the optimized score of Doench et al. (2016). DNA synthesis was adopted to construct custom sgRNA expression cassettes.

### 2.3 Biofilm assay and quantification

Wild-type and engineered strains were grown overnight in appropriate media conditions. The optical density at 600 nm ( ${\text{OD}}_{600}$ ) was measured, and cultures were diluted in 3 mL to an ${\text{OD}}_{600}$ of 0.5 in polystyrene tubes ( 12×75 mm) and grown statically at 37 °C for 24 h. The planktonic culture was collected, and its optical density at 600 nm ( ${\text{OD}}_{600}$ ) was measured. Biofilms formed at the air–liquid culture interface, adhering to the tube surface. The planktonic culture was removed by drawing it up carefully with a pipette, while the biofilm adhering to the tubes was washed once with 4 mL of 1 × PBS (Dulbecco’s), then stained with 4 mL of 20% ethanol containing 0.1% crystal violet (Merck) for 10 min. After staining, biofilms were washed twice with 1 × PBS to remove excess dye. Biofilm production was quantified using plate reader analysis, as described below.

#### 2.3.1 RNA extraction and gene expression analysis

To evaluate the repression of OmpA synthesis by CRISPRi, transcription of the target gene was analyzed as follows. Overnight bacterial cultures were processed for RNA extraction using the GRS Total RNA Kit - Bacteria (GRISP Research Solutions) following the manufacturer’s instructions. The extracted RNA was quantified using a NanoDrop spectrophotometer (Thermo Fisher Scientific). Gene expression levels were evaluated using one-step real-time PCR with the iTaq Universal SYBR Green One-Step Kit (Bio-Rad) according to the manufacturer’s protocol. Gene expression was assessed by calculating the ΔCt values, using the housekeeping gene as an internal control. Relative expression levels were then determined by the ΔΔCt method, with the *A. baumannii* wild-type (WT) ATCC #196906 strain serving as the reference sample.

### 2.4 Plate reader characterizations

#### 2.4.1 Dynamic measurement of fluorescence

Fluorescence and absorbance of recombinant strains were measured over time in a microplate reader as described by Bellato et al. (2022). In brief, bacteria from a glycerol stock were streaked on a selective LB agar plate. After an overnight incubation at 37 °C, 5 mL of selective LB medium for *E. coli* and 4 mL for *A. baumannii* was inoculated with a single colony. After incubation for 21 h at 37 °C, 200 rpm in an orbital shaker, cultures were diluted 100-fold in 200 μ L in a 96-well microplate. Given the need for additional dilution for *A. baumannii*, each inoculum was further diluted 100-fold 4 h before the experiment into 2 mL of selective medium.

Serial dilution of selective media were prepared with the inducers N-3-oxohexanoyl-l-homoserine lactone (HSL, #K3007, Sigma-Aldrich), isopropyl- β -d-1-thiogalactopyranoside (IPTG, #I1284, Sigma-Aldrich), or anhydrotetracycline (aTc, Thermo Fisher Scientific #12813196) and used in a plate to reach the desired final inducer concentration in the 0.1–500 nM, 0.05–2 mM, and 0–500 ng/mL ranges, respectively.

External wells of the plate were not used to avoid evaporation artifacts in bacterial concentration measurements. The microplate was incubated with the lid in the Varioskan™ LUX Multimode Microplate Reader (Thermo Fisher Scientific) and assayed via a kinetic cycle: continuous orbital shaking (3 mm amplitude), 5 s wait, absorbance (600 nm) measurement, fluorescence measurements, 5 min sampling time. Red and green fluorescence signals from RFP and GFP were measured at excitation wavelengths of 535 nm and 485 nm, respectively, with emission wavelengths of 620 nm and 540 nm. Control wells were also included, as described below, to measure the background signals of absorbance and fluorescence and to provide internal control references for calculating relative activity. At least three biological replicates, starting from different colonies, were assayed for each strain.

#### 2.4.2 Crystal violet quantification

To quantify the stained biofilms in the air-dried tubes, solubilization was performed using 3 mL of 30% acetic acid. The absorbance of the solubilized biofilms was measured at 592 nm (OD_592_) using the Varioskan™ LUX Multimode Microplate Reader (Thermo Fisher Scientific) and normalized to the corresponding OD_600_ of the planktonic culture.

#### 2.4.3 Data analysis

Data analysis and graphs were performed and generated using GraphPad Prism 8.0.1, Microsoft Excel, and MATLAB R2024b (MathWorks, Natick, MA). Raw absorbance and fluorescence time series acquired from microplate experiments were background-subtracted using the average signal of triplicate controls for each time point: a non-engineered strain for fluorescence and empty media for absorbance. The fluorescence outputs of recombinant strains from microplate experiments were computed in terms of steady-state RFP/ ${OD}_{600}$ and GFP/ ${OD}_{600}$ per cell in arbitrary units per cell. The average outputs in the ${\text{OD}}_{600}$ growth phase of interest (either exponential or stationary) were computed as Equations 1–3:

$$\frac{F}{OD_{600}}\left(t\right)=F\left(t\right)/OD_{600}\left(t\right),$$ (1)

$$\begin{array}{ll} & \forall OD_{600}^{\min}\leq t\leq OD_{600}^{\max}\\ {\frac{F}{OD_{600}}}^{\mathrm{ave}} & =mean\left(\frac{F}{OD_{600}}\left(t\right)\right), \end{array}$$ (2)

$${\frac{F}{OD_{6}00}}^{\mathrm{norm}}={\frac{F}{OD_{600}}}^{\mathrm{ave}}/{\frac{F}{OD_{600}}}^{\mathrm{ave,ref}},$$ (3)

with F(t) as the general fluorescent protein time series, background-subtracted, ${\frac{F}{OD_{600}}}_{\mathrm{ave}}$ as the mean of the signal over the time frame of interest, and ${\frac{F}{OD_{600}}}^{\mathrm{norm}}$ as the signal normalized on a reference strain (e.g., Bba_I13521 for RFP constitutive signal).

In particular, the stationary phase $\left(OD_{600}>0.2\right)$ of *A. baumannii* was used for the aTc-inducible system characterization, while the exponential growth phase $\left(0.02<OD_{600}<0.2\right)$ has been adopted for all the other experiments due to different behavior in steady-state reporter production (see Supplementary Figure S2,3). Data are always reported with the standard error of the mean (SEM) of the experimental replicate values, calculated as in Equations 4:

$$err=\frac{\sigma \left(\text{values}\right)}{\sqrt{\#\text{replicates}}},$$ (4)

with σ representing the standard deviation and the square root of the number of replicates in the denominator.

Statistics were computed with GraphPad Prism 10, and statistical significance was determined using the integrated one-way ANOVA tool.

## 3 Results

The values of RFP/ ${OD}_{600}$ per cell were used to determine the relative strength of 13 constitutive promoters from the Anderson library and to characterize the functioning of three inducible systems for gene expression: respectively, the HSL-, aTc-, and IPTG-inducible systems. Those three systems are well-characterized in *E. coli*, but only a few partial characterizations have been previously reported in *A. baumannii* (Jacobs et al., 2014). The measure of RFP/ ${OD}_{600}$ per cell was also used to characterize and compare the functioning of plasmids pSGAbi_I13521 and pME6032_I13521 inside both *A. baumannii* and *E. coli*. Growth data are reported in Supplementary Figure S2.

### 3.1 Two plasmid vectors

pSGAb-km is an *E. coli*–*Acinetobacter* species shuttle vector, suitable for cloning and gene expression in *A. baumannii*. It originated from the fusion of the pWH1277 natural cryptic plasmid of *A. calcoaceticus* BD413 and the *E. coli* pBR322 vector (Wang et al., 2019), with a kanamycin resistance cassette as the selection marker. The two origins of replication, *E. coli-ColE1* and *A. calcoaceticus-pWH1277*, allow replication in both E. *coli* and *Acinetobacter* spp. with an estimated copy number of 56–70 copies per chromosome in *A. baumannii* and *E. coli*.

The pME6032 vector is also a shuttle vector, suitable for gene expression in both *E. coli* and *A. baumannii*. The subsequent editing process described below led to the creation of pME6032_I13521, a plasmid carrying *blaR*, conferring resistance to ampicillin in *E. coli* and to carbenicillin in *A. baumannii*. pME6032_I13521 presents two origins of replication: p15A for *E. coli* and pVS1 for *A. baumannii* (Heeb et al., 2002).

**FIGURE 1:**
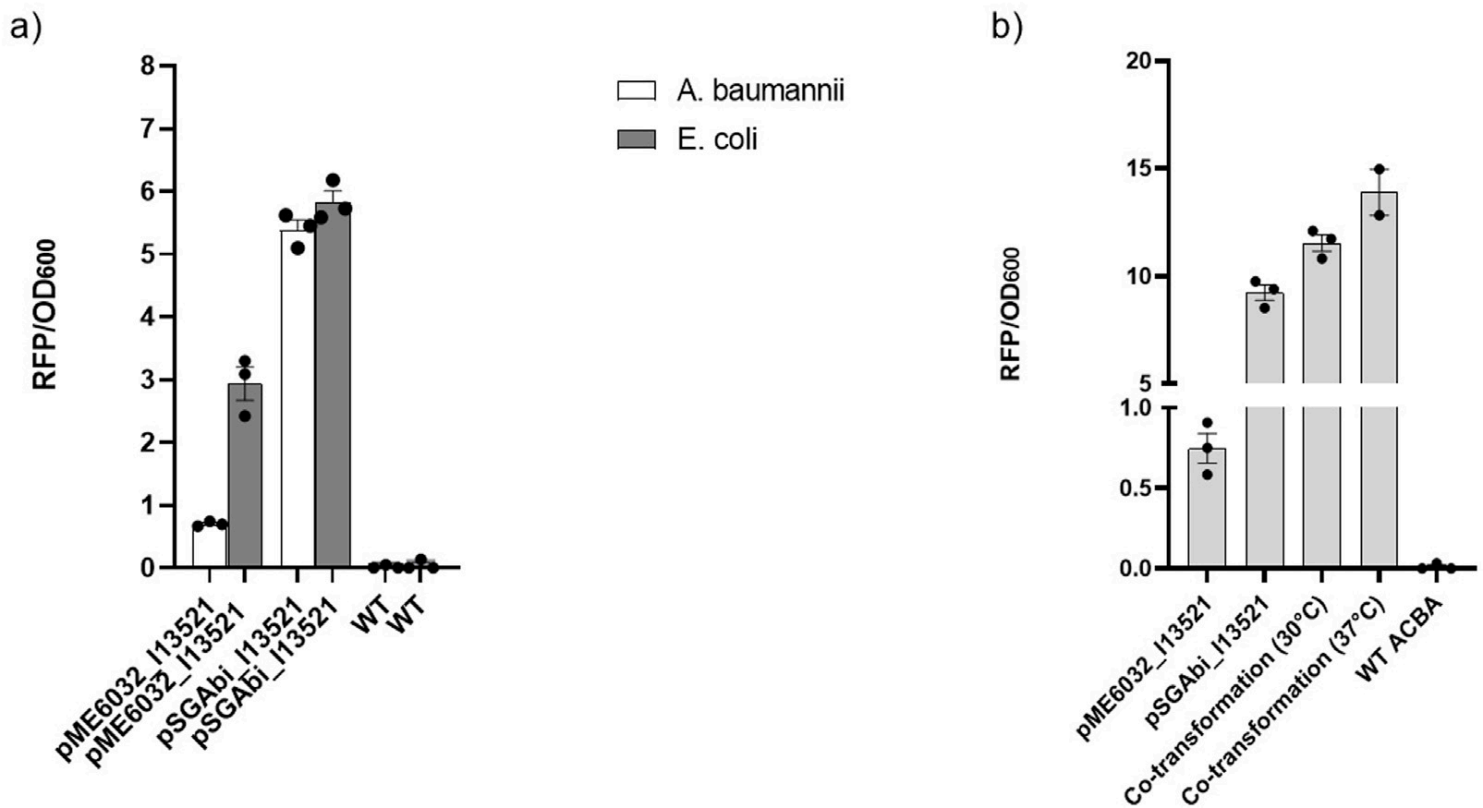
Two-plasmid system. **(a)** Values of RFP/ ${OD}_{600}$ comparing the two plasmids in *Acinetobacter baumannii* and *E. coli*, comparing also to the wild type of both strains. Data are reported as mean value with standard error over three biological replicates. **(b)** RFP/ ${OD}_{600}$ values of the co-transformed *A. baumannii* cells compared to the single transformed cells and to wild-type *A. baumannii*. Dots in the histograms represent single biological replicates and are reported along with the relative standard error intervals.

Both plasmids were modified to be compliant with the BioBrick standard RFC [10]. The maps of the two plasmids are reported in the Supplementary Figure S1. Since both plasmids carry the same constitutive expression cassette BBa_I13521, it is possible to compare the values of RFP/ ${OD}_{600}$ per cell obtained by testing the constructs in *E. coli* and *A. baumannii*. As shown in Figure 1a, the two plasmids show similar results when monitoring the fluorescence inside *E. coli*. Meanwhile a strong decrease in the RFP/ ${OD}_{600}$ values is detected when considering the expression in *A. baumannii*, reasonably due to a lower number of copy of pME6032_I13521 inside *A. baumannii*.

To test the compatibility and synergistic effect of the two plasmids, pME6032_I13521 and pSGAbi_I13521 were co-transformed into *A. baumannii*. To perform the co-transformation, cells of *A. baumannii* were transformed with DNA from both plasmids. The transformed cells were then cultured at 30 °C and 37 °C to determine which temperature better supported cell growth and protein expression for a two-plasmid system. The RFP/ ${OD}_{600}$ values are shown in Figure 1b: co-transformed cells exhibited higher RFP/ ${OD}_{600}$ values than single-plasmid transformations, with 37 °C resulting in higher values than 30 °C. These data confirm the compatibility of the two plasmids inside *A. baumannii* and demonstrate the functionality of a new two-plasmid system, which can be utilized to test different elements simultaneously within *A. baumannii* by leveraging the differences in copy number of the two plasmids.

### 3.2 Constitutive promoter library

**FIGURE 2:**
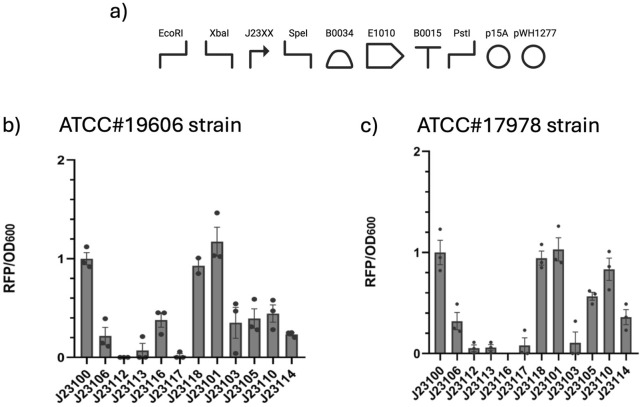
Constitutive library characterization. **(a)** Genetic circuit of the pSGAbi_J23XX_RFP set of plasmids. **(b)** Average values of RFP/ ${OD}_{600}$ values of constitutive promoters expressing BBa_E1010, normalized over promoter J23100 in ATCC #19606 . **(c)** Average values of RFP/ ${OD}_{600}$ values of constitutive promoters expressing RFP (BBa_E1010), normalized over promoter J23100 in ATCC #17978. Dots in the histograms represent single biological replicates and are reported along with the relative standard error intervals.

Different promoters from the Anderson library were cloned and tested inside *A. baumannii*. The Anderson library is a collection of 20 promoters discovered by Chris Anderson, which have already been characterized in *E. coli*, available on the Registry of Standard Biological Parts (2025). The relative strength of the promoters was evaluated by measuring the fluorescence driven by each of them. In this study, 13 promoters expressing RFP BBa_E1010 were tested. In brief, the structure of the RFP cassette is the following: Anderson promoter BBa_J23XX, RBS BBa_B0034, RFP BBa_E1010, and terminator BBa_B0010 (Figure 2a). Each expression cassette was cloned into pSGAbi (Supplementary Table S1); each plasmid was then transformed into *A. baumannii* via electroporation. The transformed cells of *A. baumannii* were tested, and the fluorescence measurements of RFP/ ${OD}_{600}$ per cell were used to determine the relative strength of each promoter: all of the promoters tested were normalized over the value of BBa_J23100. The RFP/ ${OD}_{600}$ values are shown in Figure 2b. The RFP/ ${OD}_{600}$ values show that, except for promoters BBa_J23112, BBa_J23113, and BBa_J23117, all others function inside *A. baumannii*, where BBa_J23100 and BBa_J23101 show the highest fluorescence, along with BBa_J23118.

A further inter-strain characterization was performed by analyzing the same promoter library on the *A. baumannii* laboratory strain ATCC #17978 (Figure 2c). The results align with those observed for the other strain, showing similar rankings of strengths except for the promoter BBa_J23103, which resulted in almost no expression, and the promoter BBa_J23116, which led to no colonies after several electroporation attempts. Lastly, when comparing the data on the strength of the promoters reported in the MIT Registry of Standard Biological Parts (listed in Supplementary Table S3), parts BBa_J23100, J23101, and J23118 exhibit the highest strength in both the *A. baumannii* strains and in *E. coli*. Meanwhile, BBa_J23103, which exhibits a very low relative strength in *E. coli*, shows instead a moderate relative strength in *A. baumannii* strain ATCC #19606 .

### 3.3 Inducible library

**FIGURE 3:**
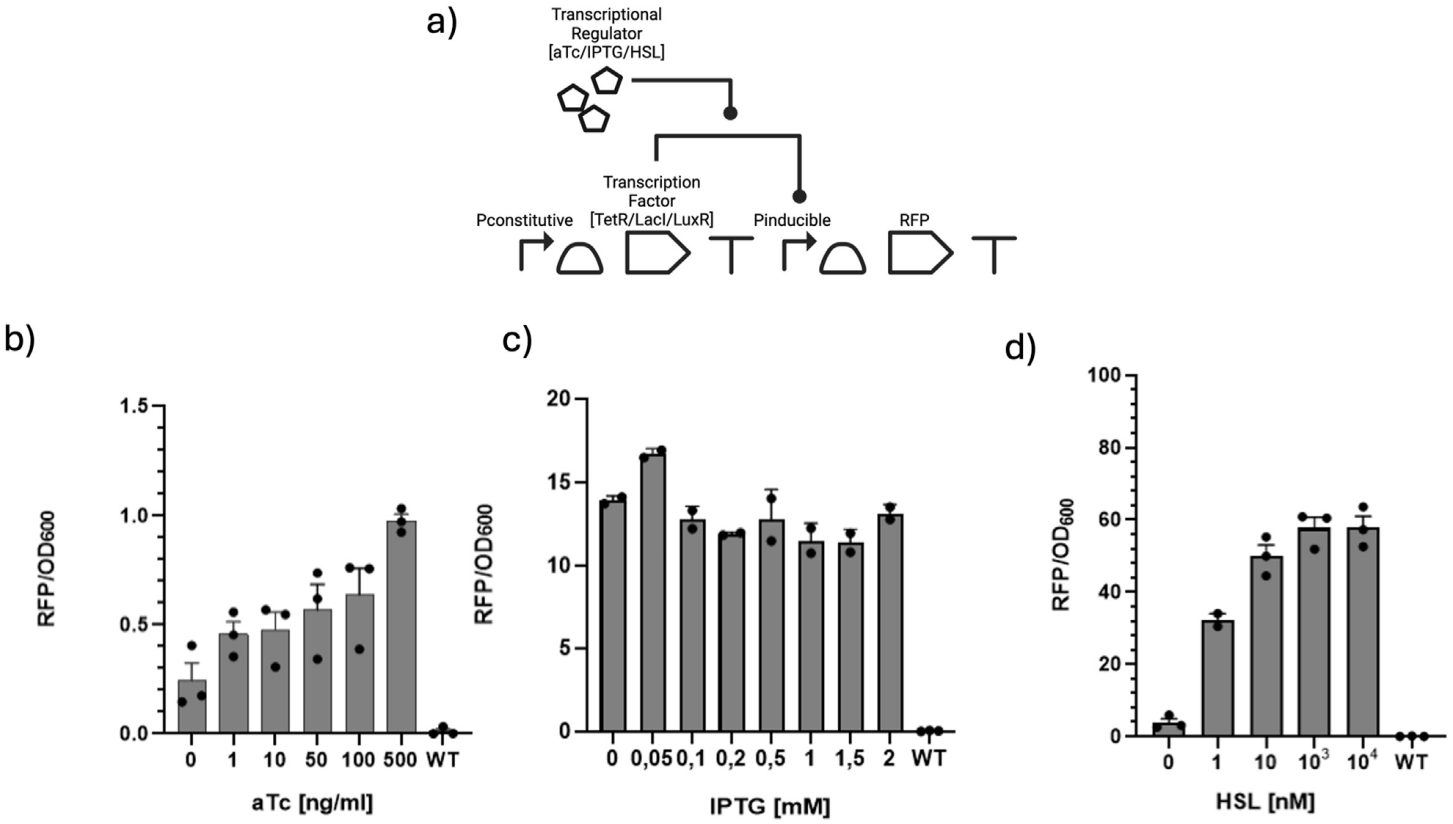
Average RFP/ ${OD}_{600}$ values for the three different inducible systems tested. **(a)** General circuit diagram with aTC inhibiting the TetR repression on pTet, IPTG relieving the LacI repression on pLac, and HSL promoting the activation of pLux by LuxR. **(b)** aTc-inducible system. **(c)** IPTG-inducible system. **(d)** HSL-inducible system. The three different systems exhibit different ranges of RFP expression, with the aTc system showing an increasing trend in the lower range of 0.0–1.5 RFP/ ${OD}_{600}$ , while HSL shows a trend in the highest range of RFP/ ${OD}_{600}$ reported, 0–80 RFP/ ${OD}_{600}$ . Dots in the histograms represent single biological replicates and are reported along with the relative standard error intervals.

Three inducible systems were tested inside *A. baumannii*: the tetracycline-inducible, the IPTG-inducible, and the HSL-inducible gene expression systems. The three genetic circuits, whose general structure is shown in Figure 3a, are briefly described below.

- The aTc-inducible gene expression system inside pSGAbi_aTc is structured as follows: the sequence of the tetracycline repressor BBa_C0040 is constitutively expressed under promoter BBa_J23118, RBS BBa_B0031, and terminator BBa_B0015; downstream is the BBa_I13521 cassette, where RFP BBa_E1010 under pTet promoter is kept silenced by TetR.
- The IPTG-inducible gene expression system inside pSGAbi_Y32_pLac_RFP contains the cassette for the production of LacI BBa_C0012 gene under promoter BBa_J23118, RBS BBa_B0034, and terminator BBa_B0015. The constitutively produced LacI protein binds to the pLac promoter BBa_R0011, keeping the expression of RFP BBa_E1010 downstream of the pLac promoter, the cassette of which is structured with RBS BBa_B0034 and terminator BBa_B0010, inhibited.
- The HSL-inducible gene expression system carried on the pSGAbi_LuxR plasmid consists of two expression cassettes. The first cassette contains the *LuxR* gene, which is expressed constitutively under the control of the BBa_R0051 promoter, with the BBa_B0030 RBS and the BBa_B1006 terminator. The second cassette includes the red fluorescent protein gene (BBa_E1010), driven by the HSL-inducible promoter BBa_R0062 (pLuxR), along with the BBa_B0034 RBS and the BBa_B0010 terminator. LuxR is a constitutively expressed transcriptional regulator that binds HSL. Upon binding HSL, the LuxR–HSL complex activates transcription from the pLuxR promoter, leading to the expression of the red fluorescent protein.

#### 3.3.1 aTc-inducible system

The aTc-inducible system was tested in *A. baumannii* by comparing the growth of cells transformed with all the aTc-inducible system with wild-type cells and with the ones transformed with only the BBa_I13521 cassette, expressing RFP constitutively under the same pTet promoter, in the absence of the TetR repressor expression cassette, and therefore without the need for an inducer. When adding aTc, the inhibition of the tetracycline repressor to the pTet promoter is removed, allowing the expression of fluorescence from RFP. To characterize the functioning of the cassette inside *A. baumannii*, several different concentrations of aTc from 0 to 500 [ng/ml] were tested.

The measurement of the ${\text{OD}}_{600}$ showed that the growth rate slowed when increasing the concentration of the inducer, with a minimum change between the concentrations from 0 to 100 [ng/ml], followed by a dramatic decrease in the growth rate when reaching 500 [ng/ml] (Supplementary Figure S2). Interestingly, even when the growth rate is slowed, the RFP/ ${OD}_{600}$ values show that increasing the concentration of inducer leads to an increasing trend in fluorescence expression of RFP (Figure 3b). Two samples were tested by inoculating the bacteria overnight in the presence of aTc—respectively 10 [ng/ml] and 100 [ng/ml]—to determine whether or not the exposure to the inducer for more time would positively affect the fluorescence expression of the bacteria or the growth (Supplementary Figure S2c). However, according to the RFP/ ${OD}_{600}$ values, incubating overnight with aTc does not enhance fluorescence expression (data not shown).

Comparing the RFP/ ${OD}_{600}$ of the cells carrying the aTc cassette to the cells carrying only BBa_I13521, therefore constitutively expressing RFP under pTet promoter, shows that *A. baumannii* cells containing the aTc cassette do not reach the same RFP/ ${OD}_{600}$ value of BBa_I13521, even with the higher concentration of inducer tested. This could occur due to the differing ranges of functionality of the two cassettes. Considering the values of RFP/ ${OD}_{600}$ over time during cell growth (Supplementary Figure S3), it is possible to determine the range of fluorescence in which the system is at its steady state (i.e., recombinant gene synthesis rate per cell is constant), and therefore in which growth phase the genetic circuit in the cell perform the best. As seen with BBa_I13521, this phenomenon typically occurs during the exponential phase of cell growth, followed by an increase in values that slows the degradation rates once the cells enter the stationary phase, typically due to a decrease in the contribution from cell division.

Regarding *A. baumannii* cells engineered with the aTc-inducible system, the RFP/ ${OD}_{600}$ values remain consistently stable throughout the entire cellular growth. Therefore, the aTc-inducible system, compared to the constitutive system, can be reliably used even in the stationary phase.

#### 3.3.2 IPTG-inducible system

*A. baumannii* cells transformed with the IPTG-inducible system were tested in the presence of IPTG to measure the difference in terms of RFP/ ${OD}_{600}$ compared to non-engineered strains of *A. baumannii*. The test was performed over a range of IPTG concentrations from 0 to 2 mM. As shown in Figure 3c, while the increase in the inducer concentration did not show any particular effect on the cell growth, the RFP/ ${OD}_{600}$ values per cell were already significantly higher than the wild type even when no inducer was added; the progressive increase in concentration of IPTG did not show any trend of the RFP/ ${OD}_{600}$ . Given such results, it is possible to assume that the LacI gene is weakly expressed under the RBS BBa_B0034 in *A. baumannii*, therefore leading to a weak repression of BBa_E1010 and higher RFP/ ${OD}_{600}$ values per cell. By plotting the RFP/ ${OD}_{600}$ over time during cell growth, it can be determined that this system is active during the exponential phase of cell growth, maintaining a considerable level of RFP/ ${OD}_{600}$ for all the different concentrations of inducer tested (Supplementary Figure S3). Since the promoter expressing LacI is already among the strongest available, we attempted to modify the RBS in a final effort to optimize the system. We switched from the RBS BBa_B0034 to BBa_B0032, which was predicted to be roughly ten times stronger based on *in silico* analysis via the De Novo DNA RBS calculator tool (Reis and Salis, 2020) set for *A. baumannii*. However, no colonies appeared after multiple attempts, indicating an excessive load on LacI synthesis in these tested genetic construct configurations. Similarly, by splitting the system into two plasmids—LacI constitutive expression cassette on pSGAbi- and LacI/IPTG-inducible RFP expression cassette in pME6032—no colonies appeared upon electroporation, probably due to overload caused by the two plasmids with high-demanding expression cassettes.

#### 3.3.3 HSL-inducible system

The HSL-inducible system was tested in *A. baumannii* strain 19,606. To assess the system’s functionality, the RFP gene was cloned downstream of the inducible promoter. When cells harboring the plasmid were treated with HSL, the molecule bound to the transcription factor LuxR, which in turn activated RFP gene expression. Different concentrations of the inducer were tested, from 0 to $10^{4}$ nM. As shown by the graph in Figure 3d, the engineered strain induced with $10^{3}$ and $10^{4}$ nM HSL showed up to a 50-fold increase in RFP fluorescence compared to the non-induced control. These data indicate that the HSL-inducible system is functional in *A. baumannii* strain and effectively drives gene expression in response to the signaling molecule.

### 3.4 CRISPR interference

#### 3.4.1 Plasmid reporter gene target

**FIGURE 4:**
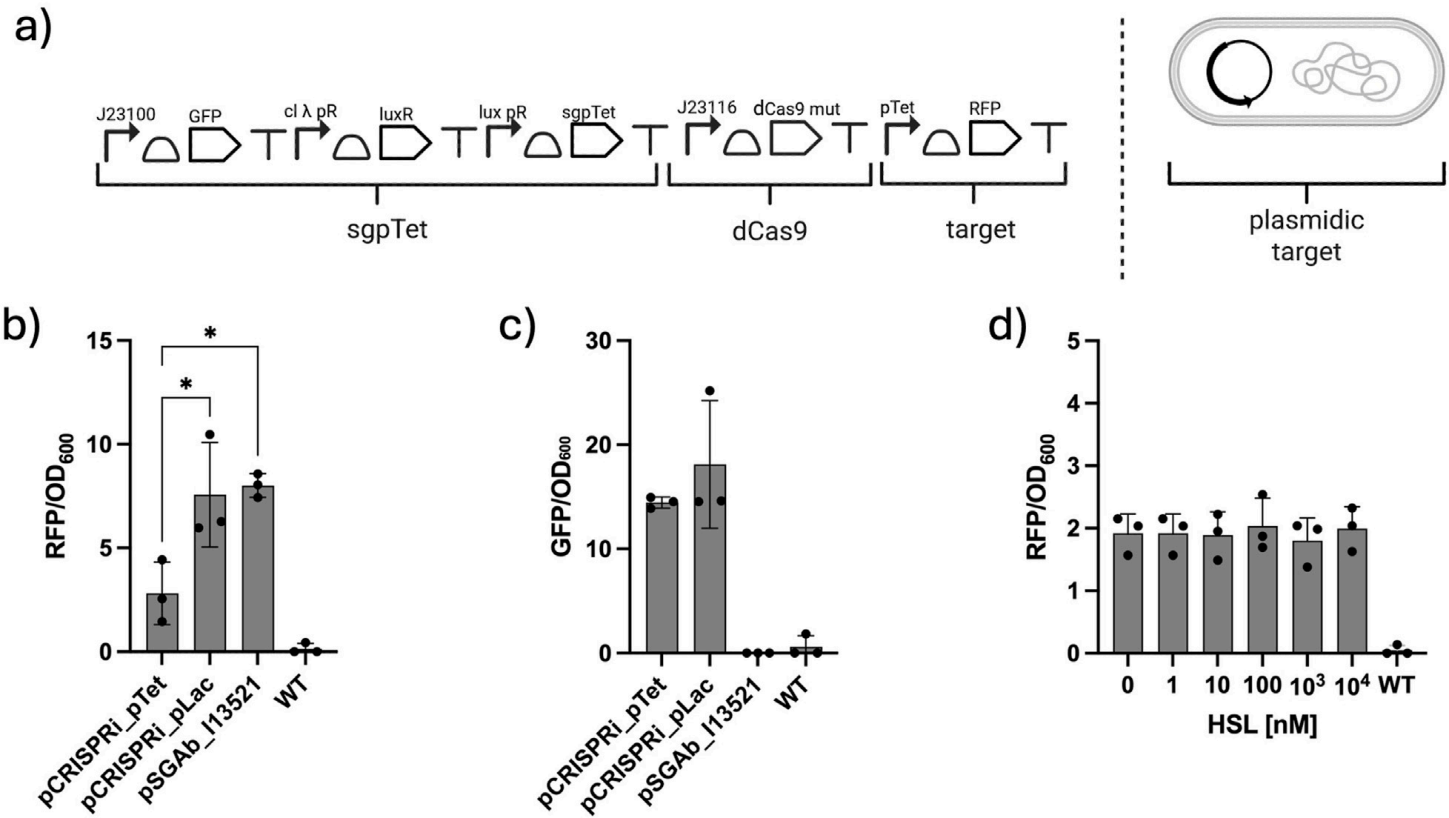
Characterization of CRISPRi on reporter gene target in *A baumannii.* **(a)** Schematics of the genetic circuit targeting the plasmid reporter. **(b)** Basal repression of the reporter gene compared with a negative control and wild-type strain. **(c)** GFP expression as a proxy of the metabolic burden in the CRISPRi expression cassettes. **(d)** Repression of the reporter gene for different inductions of the gRNA expression. Dots in the histograms represent single biological replicates and are reported along with the relative standard error intervals.

To silence a plasmid target, a RFP was targeted using a single plasmid carrying both the CRISPRi machinery and the target sequence. The pSGAbi-CRISPRi plasmid consists of a previously described HSL-inducible system regulating the expression of sgPTet, a mutagenized dCas9, and the target gene I13521 (Figure 4a.)

To evaluate the ability of the CRISPRi circuit to repress RFP, a dynamic plate reader assay was performed to measure fluorescence over 14 h. As shown in Figure 4b, the pSGAbi-CRISPRi system can repress RFP expression, though not completely, and its silencing efficiency is approximately fivefold lower than that of the control plasmids pSGAbi_I13521 and pSGAbi_pLac. The latter bears a mutagenized aspecific sgRNA targeting the promoter pLac instead of the target promoter pTet to assess off-target effects and to verify whether the CRISPRi system could specifically target the RFP. The pSGAbi-CRISPRi plasmids contain an additional GFP expression cassette to evaluate a possible burden effect caused by the large number of parts cloned into it (Pasotti et al., 2019; Bellato et al., 2022). As shown in Figure 4c, GFP expression in both pCRISPRi-pTet and pCRISPRi-pLac is equal, demonstrating that the variation in RFP does not lead to metabolic load variations.

In the pSGAbi-CRISPRi design, the sgRNA is expressed under the control of an HSL-inducible promoter, while dCas9 is constitutively expressed under the J23116 promoter. Different HSL induction conditions were tested, ranging from 0 to 10 μM, to evaluate the tunability of the implemented system. The RFP expression across all conditions was similar to that of the non-induced control. This indicates that varying HSL induction levels did not affect the engineered *A. baumannii* strain (Figure 4d). These findings suggest that the J23116 promoter upstream of *dCas9* may produce insufficient expression to enable complete repression. Thus, the repressor protein is already saturated by the leaky expression of gRNAs from the HSL-inducible system.

#### 3.4.2 Genomic AMR-associated target

**FIGURE 5:**
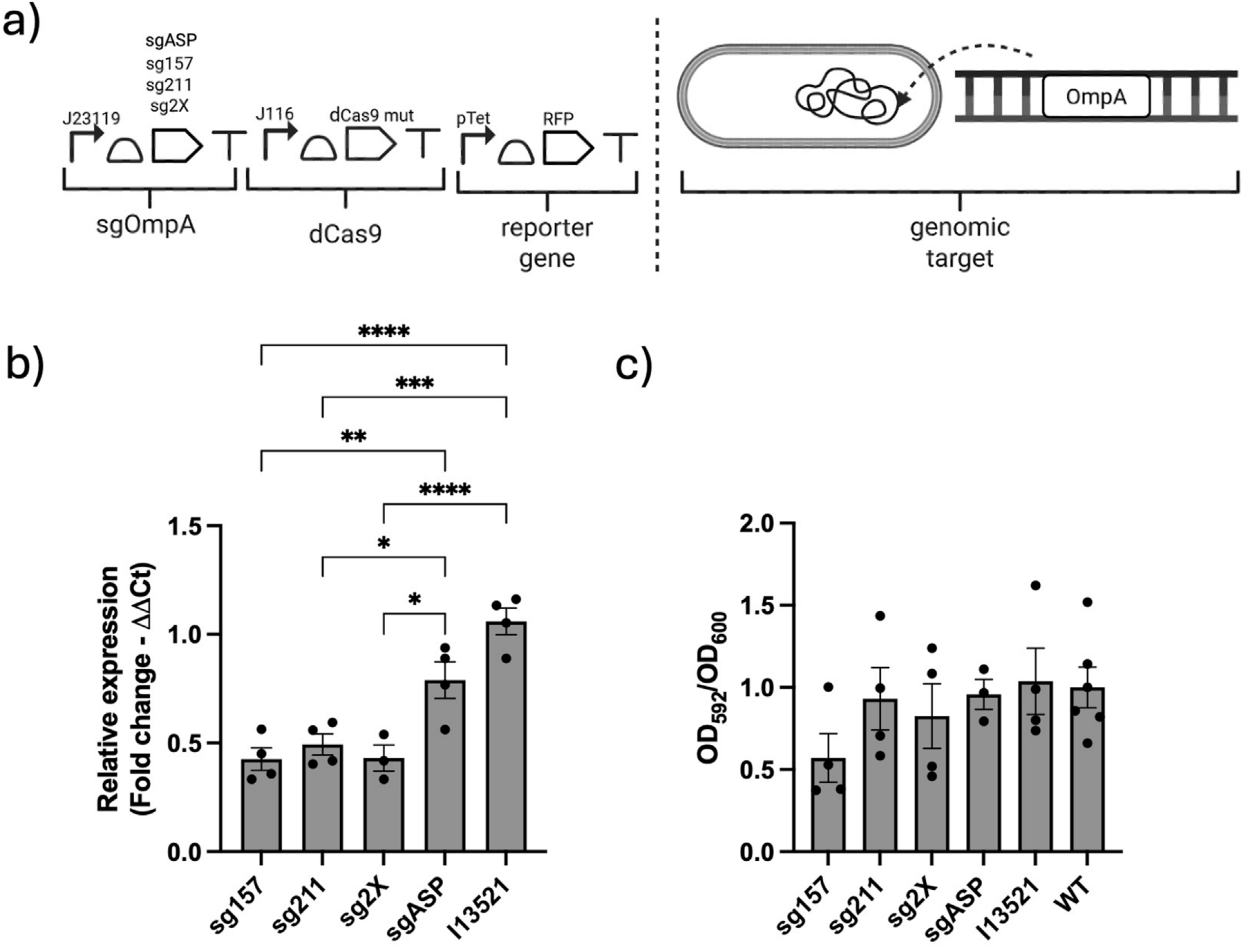
Characterization of CRISPRi on an endogenous gene in *A. baumannii*. **(a)** Schematics of the genetic circuit targeting the genomic target. **(b)** Real-time PCR expression of the target gene when targeted with different gRNAs, with negative controls. **(c)** Phenotypic outcome of the biofilm formation measured via the crystal violet assay. Dots in the histograms represent single biological replicates and are reported along with the relative standard error intervals. All data are normalized on the wild-type *A. baumannii* ATCC #19606 control strain.

Despite incomplete repression of RFP, as the system capable of exerting a strong gene expression repression, a genomic target was also tested. The *OmpA* gene, implicated in biofilm formation, was chosen as a genomic target to be silenced. Three plasmids carrying guide RNAs targeting distinct sites were evaluated (Figure 5a): one binding within 157 bp (sg157) downstream of the target gene starting codon, one targeting the 211 bp (sg211) position, and both concomitantly expressed to bind to positions simultaneously (2×).

These plasmids were tested alongside a non-targeting, nonspecific control gRNA designed to bind to the synthetic promoter pLlacO1 (Bellato et al., 2022). Biofilm formation assays were performed, and gene silencing efficiency was confirmed by measuring *ompA* mRNA expression levels (Figure 5b). The results indicated that all constructs containing targeting guides effectively reduced OmpA expression at the transcription level, despite some variability. A less obvious result was observed with the additional Crystal violet assay, as reported in Figure 5c and Supplementary Figure S4. This is likely because OmpA is related but is not the only gene responsible for biofilm formation, so phenotype analysis may yield more variable and less definitive results.

Additionally, the pSGAbi_I13521 plasmid was used as a control to determine whether the selective growth conditions were linked to the plasmid-affected biofilm formation. Compared to the wild-type strain, mRNA synthesis and biofilm formation in the pSGAbi_I13521 strain appeared similar to the WT control; however, the aspecific gRNA sgASP resulted in a slight, non-statistically significant decrease compared to the other control. This is probably due to a redistribution of translational resources linked to the metabolic burden of the CRISPRi system, which impacts growth and the production of endogenous and recombinant genes differently (Cimolato et al., 2025).

## 4 Discussion

The modification of the two plasmid vectors psGAB-km and pME6032 into pSGAbi and pME6032_I13521 led to the creation of new shuttle vectors compatible with the BioBrick RFC [10] standard for synthetic biology in *A. baumannii*. The origins of replication of pME6032_I13521 and pSGAbi_I13521 are compatible, virtually allowing the co-transformation of both vectors inside *A. baumannii*, and the difference in the copy number is suitable for tuning gene expression. Further studies will focus on optimizing protocols and tuning gene expression for the co-transformation of the two plasmids. This will be used to evaluate the CRISPRi system in one plasmid by targeting gene expression in the other.

The optimization of two different shuttle vectors for gene expression in *E. coli* and *A. baumannii* allowed the study of the functioning of both constitutive and inducible gene expression systems, which are already well-characterized in *E. coli*, inside *A. baumannii*. Testing the Anderson library of promoters inside *A. baumannii* allowed the identification of BBa_J23100, BBa_J23101, and BBa_J23118 as the stronger constitutive promoters in the library, showing a similarity with the expression inside *E. coli*. Promoters BBa_J23112, BBa_J23113, and BBa_J23117 do not seem to function inside *A. baumannii*, as with *E. coli*, while promoter BBa_J23103 appears to work better in *A. baumannii* than *E. coli*. These differences are also generally conserved across two different species of *A. baumannii*, indicating a broader applicability among various strains of the same species and thus reducing the effort needed to redesign and debug genetic circuits when migrating through different *A. baumannii* host strains.

Regarding the inducible gene expression systems, the aTc-inducible system seems to work properly inside *A. baumannii*, showing an increasing trend with the increase of inducer, despite exhibiting low RFP/ ${OD}_{600}$ values compared to the corresponding constitutive version BBa_I13521 cassette. The HSL-inducible system is well responsive inside *A. baumannii*, showing a drastic increase in RFP/ ${OD}_{600}$ even when the lowest level of HSL was added (1 [nM]) and already reaching a plateau of RFP/ ${OD}_{600}$ value of approximately 1000 [nM] of HSL.

Given the unsuccessful attempts to develop a functional IPTG-tunable expression system, this limitation of the proposed toolkit will require further analysis of the specific system in the tested strain and exploration of a broader circuit design space.

Despite the tuning of the CRISPRi system making the task challenging, consistent with the literature (Bellato et al., 2022; Frusteri Chiacchiera et al., 2025; Huang et al., 2021), the developed optimized system provides a promising and functional approach to downregulate AMR-associated gene expression, representing a valuable advance in the study of new therapeutics against this daunting challenge in medicine. It is worth noting that, while it has been consistently shown that two sgRNAs are enough to fully repress a target signal in bacterial CRISPRi systems, the repression efficiency can vary greatly depending on the binding site, especially when the sgRNA targets the coding sequence (Doench et al., 2016; Larson et al., 2013). Additional suggestions for designing such systems include introducing mismatches into the sgRNA to fine-tune repression levels, rather than exploring different targeting regions (Qi et al., 2013; Bellato et al., 2022). Importantly, using more than two sgRNAs may cause competition effects within the CRISPRi machinery, which can reduce repression efficiency (Huang et al., 2021; Zhang and Voigt, 2018). For the specific knockdown of phenotypic features, in addition to the optimization of dCas9 synthesis level as described in Huang et al. (2021), a helpful strategy would be to target different genes involved in the pathway; however, these design considerations, related directly to CRISPRi system implementation, are beyond the scope of this study. Lastly, it is worth noting that this study proposes to utilize CRISPR interference (CRISPRi) in place of traditional CRISPR for several reasons. First, traditional CRISPR systems that induce DNA double-strand breaks can apply strong selective pressure for the evolution of resistance to the CRISPR components themselves. Second, CRISPR-escape mutants, which evolve through mutations in the target gene, may still encode functional proteins with resistance functions, consequently giving rise to new resistance variants. By employing CRISPRi, which silences gene expression without permanently altering the DNA sequence, antimicrobial resistance genes can be effectively targeted while minimizing these associated risks.

## Data Availability

The original contributions presented in the study are publicly available. Genetic data for the novel plasmids developed in this study are available on GenBank with accession codes PX754615-PX754640. Further inquiries can be directed to the corresponding author.

## References

[B1] Antonelli G. Rossolini G. M. (2023). *Principi di microbiologia medica* (Edra).

[B2] Barbe V. Vallenet D. Fonknechten N. Kreimeyer A. Oztas S. Labarre L. (2004). Unique features revealed by the genome sequence of acinetobacter sp. adp1, a versatile and naturally transformation competent bacterium. Nucleic Acids Res. 32, 5766–5779. 10.1093/nar/gkh910 15514110 PMC528795

[B3] Bellato M. Frusteri Chiacchiera A. Salibi E. Casanova M. De Marchi D. Castagliuolo I. (2022). Crispr interference modules as low-burden logic inverters in synthetic circuits. Front. Bioeng. Biotechnol. 9, 743950. 10.3389/fbioe.2021.743950 35155399 PMC8831695

[B4] Benchling, Inc (2025). Benchling: cloud-based platform for life sciences r&d. Available online at: https://www.benchling.com (Accessed July 02, 2025).

[B5] Biswas I. Rather P. N. (2019). *Acinetobacter baumannii: methods and Protocols*, vol. 1946 of *methods in Molecular Biology* (New York, NY: Springer New York). 10.1007/978-1-4939-9118-1

[B6] Brychcy M. Kokodynski A. Lloyd D. Godoy V. G. (2023). Aspflex: molecular tools to study gene expression and regulation in acinetobacter baumannii. ACS Synth. Biol. 12, 2773–2777. 10.1021/acssynbio.3c00167 37587063 PMC10621034

[B7] Cheng G. (2019). Selection and dissemination of antimicrobial resistance in agri-food production. Antimicrob. Resist. and Infect. Control 8, 1–13. 10.1186/s13756-019-0563-0 31649815 PMC6805589

[B8] Christaki E. Marcou M. Tofarides A. (2020). Antimicrobial resistance in bacteria: mechanisms, evolution, and persistence. J. Mol. Evol. 88, 26–40. 10.1007/s00239-019-09914-3 31659373

[B9] Cimolato C. Selvaggio G. Marchetti L. Giordano G. Schenato L. Bellato M. (2025). Quorum sensing model structures inspire the design of quorum quenching strategies. IEEE Trans. Mol. Biol. Multi-Scale Commun. 11, 201–217. 10.1109/TMBMC.2025.3554671

[B10] Doench J. G. Fusi N. Sullender M. Hegde M. Vaimberg E. W. Donovan K. F. (2016). Optimized sgrna design to maximize activity and minimize off-target effects of crispr-cas9. Nat. Biotechnol. 34, 184–191. 10.1038/nbt.3437 26780180 PMC4744125

[B11] Frusteri Chiacchiera A. Casanova M. Bellato M. Piazza A. Migliavacca R. Batt G. (2025). Harnessing crispr interference to resensitize laboratory strains and clinical isolates to last resort antibiotics. Sci. Rep. 15, 261. 10.1038/s41598-024-81989-5 39747289 PMC11696610

[B12] Ghavami S. Pandi A. (2021). Crispr interference and its applications. Prog. Mol. Biol. Transl. Sci. 180, 123–140. 10.1016/bs.pmbts.2021.01.007 33934834

[B13] Heeb S. Blumer C. Haas D. (2002). Regulatory rna as mediator in gaca/rsma-dependent global control of exoproduct formation in pseudomonas fluorescens cha0. J. Bacteriol. 184, 1046–1056. 10.1128/jb.184.4.1046-1056.2002 11807065 PMC134805

[B14] Holmes A. H. Moore L. S. P. Sundsfjord A. Steinbakk M. Regmi S. Karkey A. (2016). Understanding the mechanisms and drivers of antimicrobial resistance. Lancet 387, 176–187. 10.1016/S0140-6736(15)00473-0 26603922

[B15] Huang H. Bellato M. Qian Y. Cardenas P. Pasotti L. Magni P. (2021). dcas9 regulator to neutralize competition in crispri circuits. Nat. Commun. 12, 1692. 10.1038/s41467-021-21772-6 33727557 PMC7966764

[B16] Jacobs A. C. Thompson M. G. Gebhardt M. Corey B. W. Yildirim S. Shuman H. A. (2014). Genetic manipulation of acinetobacter baumannii. Curr. Protoc. Microbiol. 35, 6G.2.1–6G.2.11. 10.1002/9780471729259.mc06g02s35 25367274

[B17] Knight F. (2007). “Draft standard for biological parts – BioBrick assembly standard (RFC 10),”. Cambridge, MA.

[B18] Larson M. H. Gilbert L. A. Wang X. Lim W. A. Weissman J. S. Qi L. S. (2013). Crispr interference (crispri) for sequence-specific control of gene expression. Nat. Protoc. 8, 2180–2196. 10.1038/nprot.2013.132 24136345 PMC3922765

[B19] Lucidi M. Runci F. Rampioni G. Frangipani E. Leoni L. Visca P. (2018). New shuttle vectors for gene cloning and expression in multidrug-resistant acinetobacter species. Antimicrob. Agents Chemother. 62, e02480-17. 10.1128/AAC.02480-17 29339383 PMC5913964

[B20] Magni P. (2025). Bioinformatics laboratory – university of pavia. Available online at: http://lab-bioinfo.unipv.it/index.php/en/(Accessed July 02, 2025).

[B21] Mancuso G. Midiri A. Gerace E. Biondo C. (2021). Bacterial antibiotic resistance: the most critical pathogens. Pathogens 10, 1310. 10.3390/pathogens10101310 34684258 PMC8541462

[B22] Montalti M. Soldà G. Capodici A. Di Valerio Z. Gribaudo G. La Fauci G. (2022). Antimicrobial resistance (amr) in Italy over the past five years: a systematic review. Biologics 2, 151–164. 10.3390/biologics2020012

[B23] O’Neill J. (2016). “Tackling drug-resistant infections globally: final report and recommendations,”. Review on Antimicrobial Resistance. London: Wellcome Trust.

[B24] Pasotti L. Bellato M. Casanova M. Zucca S. Cusella De Angelis M. Magni P. (2017). Re-using biological devices: a model-aided analysis of interconnected transcriptional cascades designed from the bottom-up. J. Biol. Eng. 11, 50. 10.1186/s13036-017-0090-3 29255481 PMC5729246

[B25] Pasotti L. Bellato M. Politi N. Casanova M. Zucca S. Cusella De Angelis M. (2019). A synthetic close-loop controller circuit for the regulation of an extracellular molecule by engineered bacteria. IEEE Trans. Biomed. Circuits Syst. 13, 248–258. 10.1109/TBCAS.2018.2883350 30489274

[B26] Pendleton S. Davidson P. M. (2017). “Microbial resistance to antimicrobials,” in Microbial control and food preservation (Springer), 173–198. 10.1007/978-1-4939-7556-3_9

[B27] Prestinaci F. Pezzotti P. Pantosti A. (2015). Antimicrobial resistance: a global multifaceted phenomenon. Pathogens Glob. Health 109, 309–318. 10.1179/2047773215Y.0000000030 26343252 PMC4768623

[B28] Qi L. S. Larson M. H. Gilbert L. A. Doudna J. A. Weissman J. S. Arkin A. P. (2013). Repurposing crispr as an rna-guided platform for sequence-specific control of gene expression. Cell 152, 1173–1183. 10.1016/j.cell.2013.02.022 23452860 PMC3664290

[B29] Registry of Standard Biological Parts (2025). Main page – igem parts registry. Available online at: https://parts.igem.org/Main_Page (Accessed July 02, 2025).

[B30] Reis A. C. Salis H. M. (2020). An automated model test system for systematic development and improvement of gene expression models. ACS Synth. Biol. 9, 3145–3156. 10.1021/acssynbio.0c00394 33054181

[B31] Scientifics T. F. (2025). Geneart™ gibson assembly hifi master mix. Available online at: https://www.thermofisher.com/order/catalog/product/A46627 (Accessed July 9, 2025).

[B32] Shetty R. P. Endy D. Knight T. F. J. (2008). Engineering biobrick vectors from biobrick parts. J. Biol. Eng. 2, 5. 10.1186/1754-1611-2-5 18410688 PMC2373286

[B33] Uni-Padua-IT iGEM Team (2023). Uni-padua-it – igem 2023. Available online at: https://2023.igem.wiki/uni-padua-it/index.html (Accessed July 02, 2025).

[B34] Wang Y. Wang Z. Chen Y. Hua X. Yu Y. Ji Q. (2019). A highly efficient crispr-cas9-based genome engineering platform in acinetobacter baumannii to understand the h2o2-sensing mechanism of oxyr. Cell Chem. Biol. 26, 1732–1742.e5. 10.1016/j.chembiol.2019.09.003 31548010

[B35] World Health Organization (2021). Antimicrobial resistance. Available online at: https://www.who.int/news-room/fact-sheets/detail/antimicrobial-resistance.Onlinefactsheet.

[B36] Zhang S. Voigt C. A. (2018). Engineered dcas9 with reduced toxicity in bacteria: implications for genetic circuit design. Nucleic Acids Res. 46, 11115–11125. 10.1093/nar/gky884 30289463 PMC6237744

