# Supplementary material for: A synthetic biology toolkit for rationally designing genetic circuits in Acinetobacter baumannii
Source: Front Syst Biol. 2026 Jan 15;5:1668595. doi: 10.3389/fsysb.2025.1668595 (PMC12852451; doi:10.3389/fsysb.2025.1668595)
Supplement: Supplementary file 1 [file Supplementaryfile1.pdf]

frontiers

Supplementary Material

1 SUPPLEMENTARY DATA

1.1 Synthesized parts

Double-strand DNA cloned into pSGAb-km for RFC 10 compatibility
`gtcaggAAGCTTGTTTCTTCGAATTCGCGGCCGCTTCTAGAGttgacagctagctcagtcctaggtataa`
`tgctagcTACTAGTAGCGGCCGCTGCAGGAAGAAACGAGCTCgtcagg`

2 SUPPLEMENTARY TABLES

Name	Source	Usage
pSGAb−km	Addgene #121999	shuttle vector for *A. baumannii* and *E. coli*
pSGAbi	Gibson Assembly	pSGAb−km with ColE1 *E. coli* ORI replaced with p15a ORI
pSB3K3	2009 iGEM Registry Distribution	origin of replication
pSB1A2_I13521	2009 iGEM Registry Distribution	expression of RFP under pTet promoter
pSGAbi_I13521	pSB1A2_I13521	expression of RFP under pTet promoter in shuttle vector
pSGAbi−LuxR	pE47	assessing inducible HSL system functionality in *A. baumannii*
pE47	Pasotti et al. (2017)	cloning HSL−inducibile system in pSGAb (EcoRI−PstI)
pSB3K3_dCas9	Bellato et al. (2022)	mutagenize EcoRI site in dCas9 sequence
pSB3K3_dCas9mut	pSB3K3_dCas9	express BioBrick-compatible dCas9
pAEgpTtet	Bellato et al. (2022)	cloning insert HSL−inducible sgpTet in the pSGAb vector
pSGAbi_CRISPRi	pAEgpTet+pSB3K3_dCas9mut+pSB1A2_I13521	CRISPRi characterization in *A. baumannii*
pAEgpLac	Bellato et al. (2022)	replacing sgpTet with sgpLac in pSGAbi vector
pSGAbi_CRISPRi_asp	pSGAb_CRISPRi+pAEgpLac	CRISPRi control in *A. baumannii* with aspecifc guide
pSGAbi_sg157	sg157+pSB3K3_dCas9mut+pSB1A2_I13521	silencing OmpA in *A. baumannii* with 157 position-gRNA
pSGAbi_sg211	sg211+pSB3K3_dCas9mut+pSB1A2_I13521	silencing OmpA in *A. baumannii* with 211 position-gRNA
pSGAbi_2X	sg157+sg211+pSB3K3_dCas9mut+pSB1A2_I13521	silencing OmpA in *A. baumannii* with two gRNA at once
pSBJ23114_RFP	2009 iGEM Registry Distribution	extracting RFP cassette
pSGAbi_J23114_RFP	This work	evaluating promoter strength in *A. baumannii*
pSBJ23103_RFP	2009 iGEM Registry Distribution	extracting RFP cassette
pSGAbi_J23103_RFP	This work	evaluating promoter strength in *A. baumannii*
pSBJ23105_RFP	2009 iGEM Registry Distribution	extracting RFP cassette
pSGAbi_J23105_RFP	This work	evaluating promoter strength in *A. baumannii*
pSBJ23113_RFP	2009 iGEM Registry Distribution	extracting RFP cassette
pSGAbi_J23113_RFP	This work	evaluating promoter strength in *A. baumannii*
pSBJ23101_RFP	2009 iGEM Registry Distribution	extracting RFP cassette
pSGAbi_J23101_RFP	This work	evaluating promoter strength in *A. baumannii*
pSBJ23110_RFP	2009 iGEM Registry Distribution	extracting RFP cassette
pSGAbi_J23110_RFP	This work	evaluating promoter strength in *A. baumannii*
pSBJ23116_RFP	2009 iGEM Registry Distribution	extracting RFP cassette
pSGAbi_J23116_RFP	This work	evaluating promoter strength in *A. baumannii*
pSBJ23100_RFP	2009 iGEM Registry Distribution	extracting RFP cassette
pSGAbi_J23100_RFP	This work	evaluating promoter strength in *A. baumannii*
pSBJ23106_RFP	2009 iGEM Registry Distribution	extracting RFP cassette
pSGAbi_J23106_RFP	This work	evaluating promoter strength in *A. baumannii*
pSBJ23112_RFP	2009 iGEM Registry Distribution	extracting RFP cassette
pSGAbi_J23112_RFP	This work	evaluating promoter strength in *A. baumannii*
pSBJ23113_RFP	2009 iGEM Registry Distribution	extracting RFP cassette
pSGAbi_J23113_RFP	This work	evaluating promoter strength in *A. baumannii*
pSBJ23117_RFP	2009 iGEM Registry Distribution	extracting RFP cassette
pSGAbi_J23117_RFP	This work	evaluating promoter strength in *A. baumannii*
pSBJ23118_RFP	2009 iGEM Registry Distribution	extracting RFP cassette
pSGAbi_J23118_RFP	This work	evaluating promoter strength in *A. baumannii*
pSB1A2_I13507	2009 iGEM Registry Distribution	cloning I13507 downstream different promoters
pSGAb_I13507	pSB1A2_I13507	cloning I13507 downstream different inducible systems in *A. baumannii*
pME6032	Heeb et al. (2002)	shuttle vector for *A. baumannii* and *E. coli*
pME6032_blaR	This work	pME6032 edited to change original tetracycline resistance with a *blaR* gene
pME6032_I13521	This work	pME6032_blaR edited to insert BBa_I13521 with flanking RFC[10] prefix and suffix
pSB4C5_ATC	Pasotti et al. (2017)	extract aTc-inducible RFP cassette to be cloned in pSGAbi
pSGABi_aTc	This work	aTc-inducible RFP expression system inside *A. baumannii*
Y32_pLac	Pasotti et al. (2019)	assemble Y32_pLac_RFP by cloning RFP dowstream pLac
Y32_pLac_RFP	This work	IPTG-inducible RFP expression under pLac
pSGAbi_Y32_pLac_RFP	This work	IPTG-inducible RFP expression system in *A. baumannii*

Table S1. List of plasmids used and generated in this study

Name	Sequence 5'-3'	Usage
FW pSGAb backbone	caagaagatcatcttattaagaaGATCCTTTGATCTTTTCTACGGGGTCTG	amplyfing pSGAb backboneexept the ORI
RV pSGAb backbone	agccagtatacactccgctaAACGCCAGCAACGCGGCCTT	amplyfing pSGAb backboneexept the ORI
FW pSB3K3 ori	aaggccgcgttgctggcgttTAGCGGAGTGTATACTGGCT	amplyfing p15a ORI
RV pSB3K3 ori	gaaaagatcaaaggatcttcTTAATAAGATGATCTTCTTGAGATCG	amplyfing p15a ORI
sg175_OmpA	aataaatcgtcttgtaactc	sgRNA design on OmpA in *A. baumannii*
sg211_OmpA	tcagcttcgaaacctaacca	sgRNA design on OmpA in *A. baumannii*
FW_sg_157_OmpA_ACBA	tgccacctgacgtctaaga	sg157 OmpA amplyfing primers
RV_sg157_OmpA_ACBA	gctcactcaaaggcggtaa	sg157 OmpA amplyfing primers
FW_sg211_OmpA_ACBA	tgccacctgacgtctaaga	sg211 OmpA amplyfing primers
RV_sg211_OmpA_ACBA	gctcactcaaaggcggtaa	sg211 OmpA amplyfing primers
FW_prefix_insertion	CGGCCGCTTCTAGAGgcatgcaagctttccctatag	mutagenesis to add EcoRI and XbaI sites
RV_prefix_insertion	CGAATTCGAAGAAACactggccgtcgttttaca	mutagenesis to add EcoRI and XbaI sites
FW_suffix_insertion	CTGCAGGAAGAAACgcatgcaagctttccctatag	mutagenesis to add SpeI and PstI sites
RV_suffix_insertion	CGGCCGCTACTAGTActctagaagcggccgcgaat	mutagenesis to add SpeI and PstI sites
Mut-J116-109 FW	cctagggactgtgctagctaCTAGA	mutagenesis to replace J23116 promoter upstream dCas9 with J23109
Mut-J116-109 RV	actgagctagctgtaaactctagaag	mutagenesis to replace J23116 promoter upstream dCas9 with J23109
FW_seq_pSGAb	gcaactggtctattttcctc	sequencing pSGAb upstream the cloning site
RV_seq_pSGAb	gcaacgcaattaatgtgagt	sequencing pSGAb downstream the cloning site
RV_mut_sgpLac	caattgttatattcgactataacaaaccattttctt	replace sgpTet with sgpLac
FW_mut_sgpLac	acattgtgagGTTTTAGAGCTAGAAATAGCAAGTTAAAA	replace sgpTet with sgpLac
FW_seq_pLacmut	caatgcgcaaatgaaactc	sequencing pSGAb with sgpLac
RV_seq_pLacmut	gatgatgatgatgatggtcg	sequencing pSGAb with sgpLac
OmpA Forward	TCTTGGTGGTCACTTGAAGC	evaluate expression of OmpA via rtPCR
Ompa Reverse	ACTCTTGTGGTTGTGGAGCA	evaluate expression of OmpA via rtPCR
FW_rpoB_RT_ACBA	GTTGCTGAAGAAGAAGCTGCTG	evaluate expression of rpoB via rtPCR
RV_rpoB_RT_ACBA	ACTGTACCCATTTCACGCATGTA	evaluate expression of rpoB via rtPCR
pME6032 FWD	aaacaaataggggttccgcgtcgtggaaacgataggccgctctaga	amplification of pME6032 plasmid removing TetR gene.
pME6032 REV	cctcactgattaagcattggtcaggaccgctgccggagcg	amplification of pME6032 plasmid removing TetR gene.
pTZ19R iGEMized FWD	cgctccggcagcggtcctgaccaatgcttaatcagtgaggc	amplification of BlaR gene from pTZ19R
pTZ19R iGEMized RV	gagcggcctatcgtttccacgacgcggaacccctatttgttt	amplification of BlaR gene from pTZ19R
FW_Ramp_A	tgtttcctgtgtgaaattgttatccgc	amplification of pME6032_blaR.
RV_Ramp_A	gctccaaggatcgggccttgatgtta	amplification of pME6032_blaR.
I13521_FW_Gibson_A	caaggcccgatccttggagcaagtgccacctgacgtctaagaaac	amplification of I13521 cassette with flanking prefix and suffix sequences.
I13521_RV_Gibson_A	acaatttcacacaggaaacaattaccgcctttgagtgagctgata	amplification of I13521 cassette with flanking prefix and suffix sequences.

Table S2. Primers list. Primers adopted for PCR in Gibson assembly, mutagenesis, and sequencing.

Bba_J23	*A. baumannii ATCC#19606*	*A. baumannii ATCC#17978*	*E. coli*
100	1	1	1
106	0.22	0.32	.47
112	0	0.02	0
113	0	0.05	0.01
116	0.38	N.A	0.16
117	0	0.06	0.06
118	0.93	0.94	0.56
101	1.17	1.03	0.7
103	0.35	0	0.01
105	0.39	0.56	0.24
110	0.44	0.83	0.33
114	0.23	0.36	0.1

Table S3. Comparison of promoter strength normalized on BBa_J23100 in *A. baumannii* and *E. coli*. Data for *E. coli* are reported in the MIT Registry of Standard Biological Parts.

3 SUPPLEMENTARY FIGURES

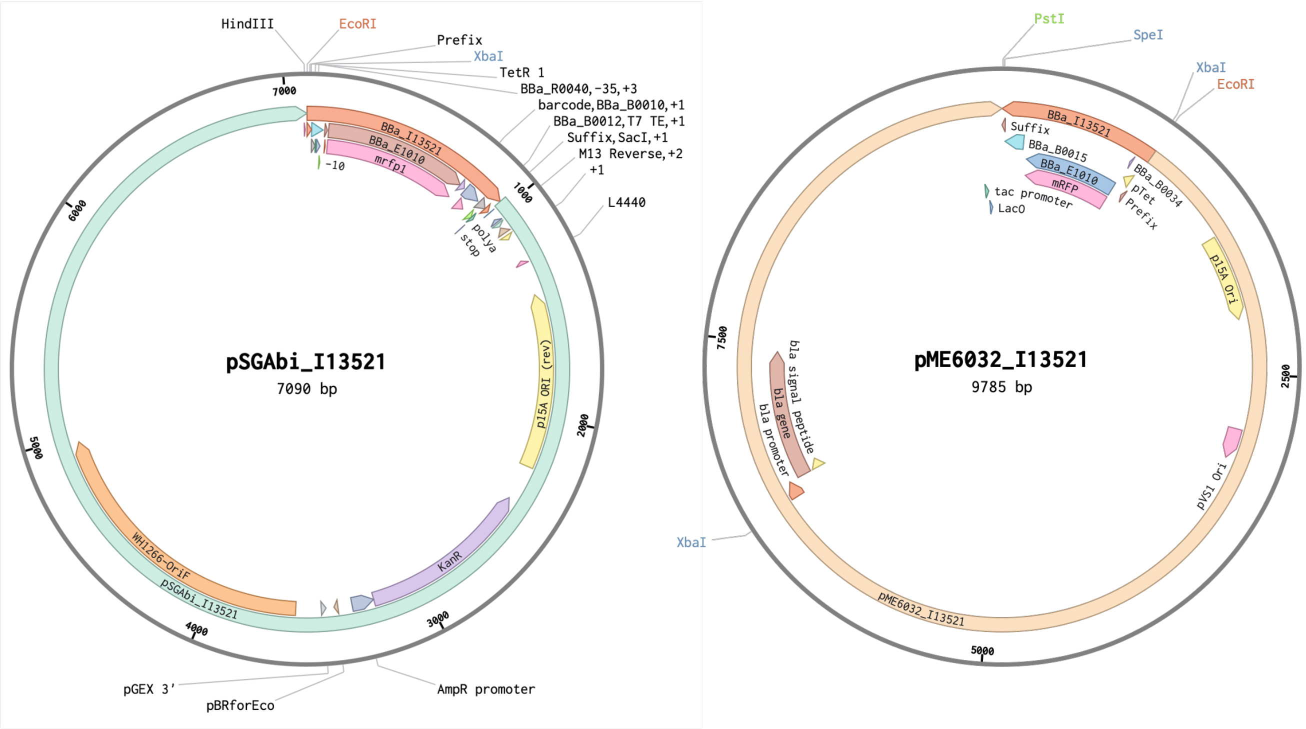

Figure S1. Two plasmid system. Maps of the two plasmid backbones developed in this study bearing BBa_I13521 RFP expression cassette as insert.

REFERENCES

Bellato, M., Frusteri Chiacchiera, A., Salibi, E., Casanova, M., De Marchi, D., Castagliuolo, I., et al. (2022). Crispr interference modules as low-burden logic inverters in synthetic circuits. *Frontiers in Bioengineering and Biotechnology* 9, 743950. doi:10.3389/fbioe.2021.743950

Heeb, S., Blumer, C., and Haas, D. (2002). Regulatory rna as mediator in gaca/rsma-dependent global control of exoproduct formation in pseudomonas fluorescens cha0. *J Bacteriol.* 184, 1046–1056. doi:https://doi.org/10.1128/jb.184.4

Pasotti, L., Bellato, M., Casanova, M., Zucca, S., Cusella De Angelis, M., and Magni, P. (2017). Re-using biological devices: a model-aided analysis of interconnected transcriptional cascades designed from the bottom-up. *J Biol Eng* 11. doi:10.1186/s13036-017-0090-3

Pasotti, L., Bellato, M., Politi, N., Casanova, M., Zucca, S., Cusella De Angelis, M., et al. (2019). A synthetic close-loop controller circuit for the regulation of an extracellular molecule by engineered bacteria. *IEEE Trans Biomed Circuits Syst* 13, 248–258. doi:10.1109/TBCAS.2018.2883350

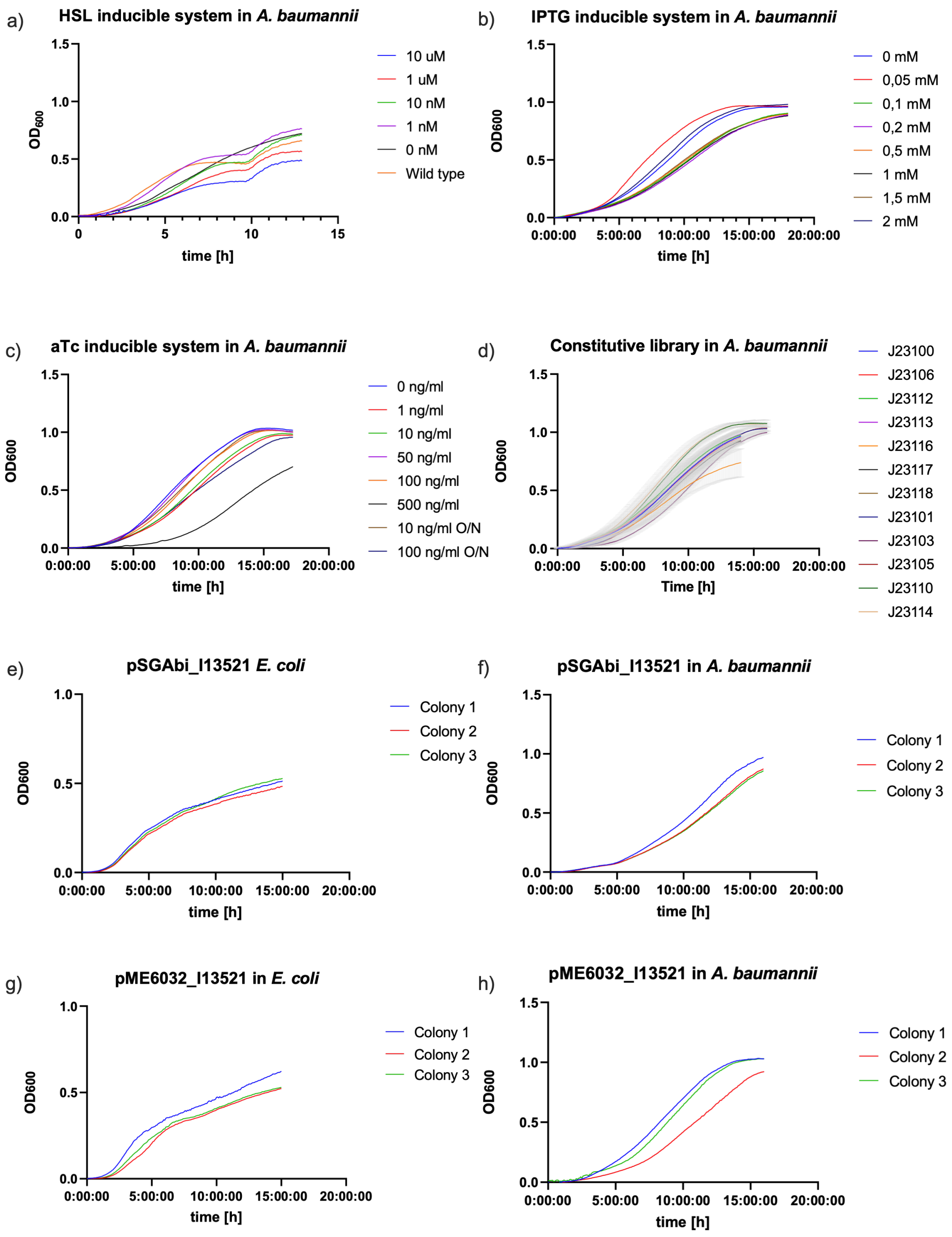

Figure S2. Growth curves. OD_{600} of the constructs tested in this study: a) average curves for three replicates per induction of the HSL-inducible system b) average curves for three replicates per induction of the IPTG-inducible system c) average curves for three replicates per induction of the aTc-inducible system d) Anderson library, grey shades represent the standard error on three replicates per construct e) Three replicates of pSGABi plasmid bearing the BBa_I13521 RFP expression cassette in *E.coli* f)Three replicates of pSGABi plasmid bearing the BBa_I13521 RFP expression cassette in *A. baumannii* g)Three replicates of pME6032 plasmid bearing the BBa_I13521 RFP expression cassette in *E.coli* h)Three replicates of pME6032 plasmid bearing the BBa_I13521 RFP expression cassette in *A. baumannii*

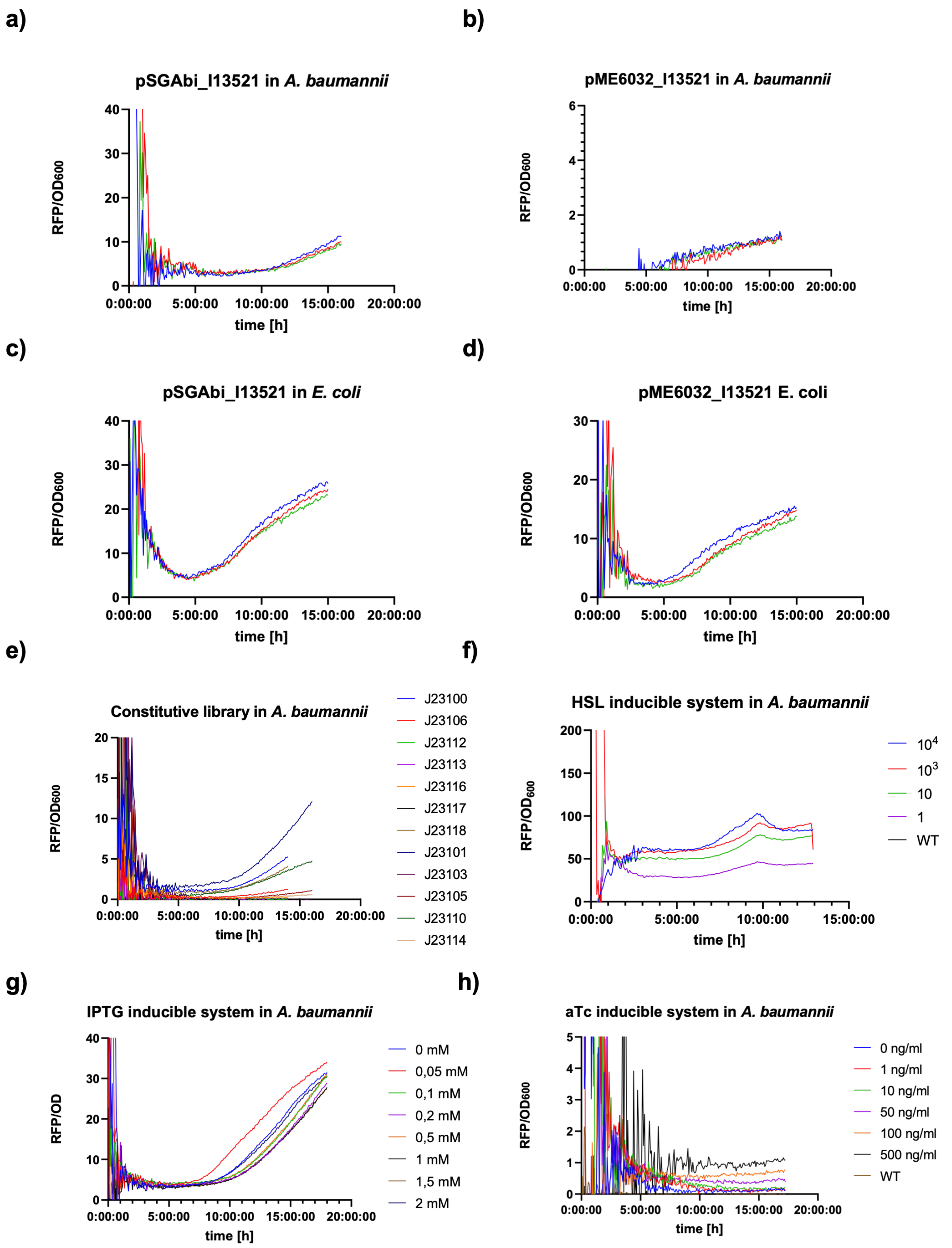

Figure S3. RFP per cell synthesis curves. RFP/OD_{600} of the constructs tested in this study: a)Three replicates of pSGABi plasmid bearing the BBa_I13521 RFP expression cassette in *A. baumannii* b) Three replicates of pME6032 plasmid bearing the BBa_I13521 RFP expression cassette in *A. baumannii* c) Three replicates of pSGABi plasmid bearing the BBa_I13521 RFP expression cassette in *E.coli* d) Three replicates of pME6032 plasmid bearing the BBa_I13521 RFP expression cassette in *E.coli* e) curves for the Anderson library, one per replicate per construct f) Example curves, one per replicate per induction, of the HSL-inducible system g) Example curves, one per replicate per induction, of the IPTG-inducible system h) Example curves, one per replicate per induction, of the aTc-inducible system.

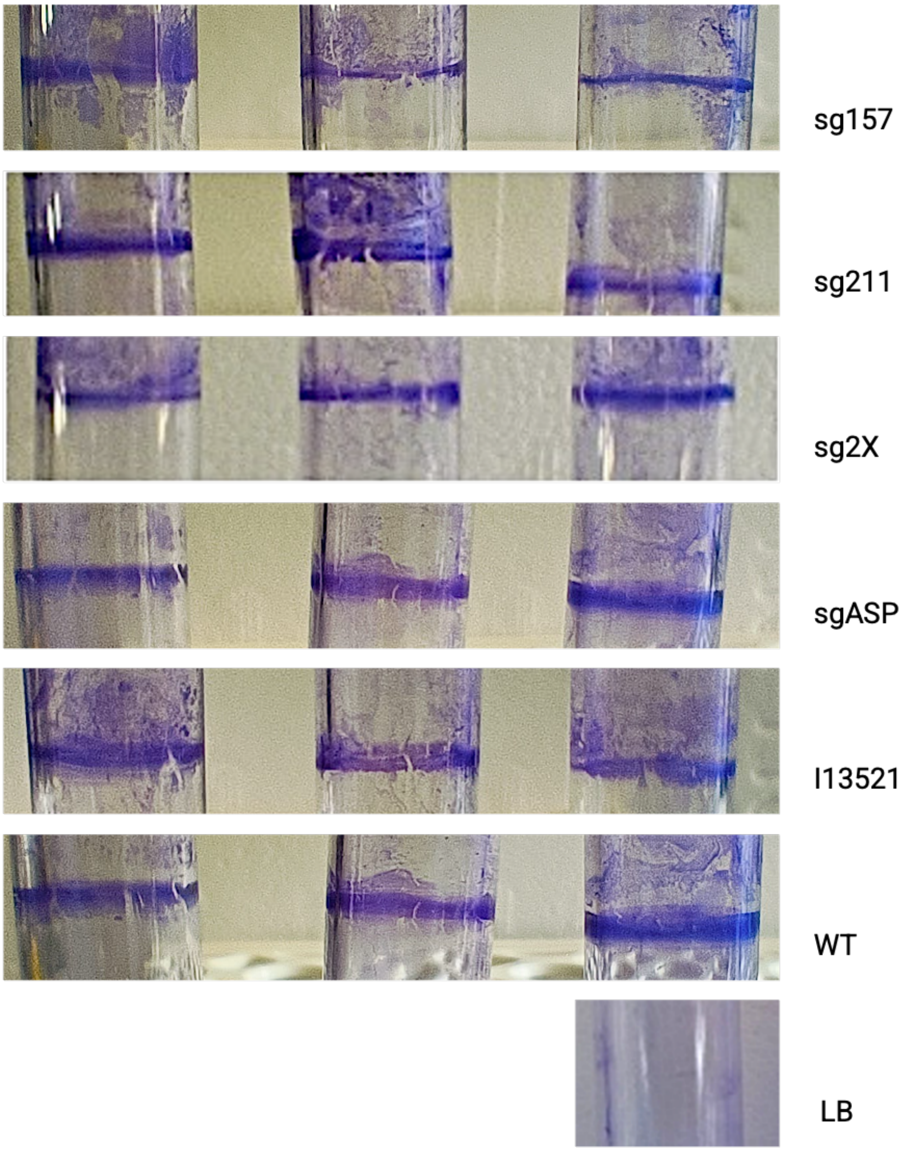

Figure S4. Crystal violet-stained tubes showing biofilm formation. Zoomed region of the tubes after staining and wash steps, highlighting biofilm formation, for the different tested strains. Wild-type (WT) ATCC#19606 strain and pure LB were used as controls.
